# Human brain endothelial cells endeavor to immunoregulate CD8 T cells via PD-1 ligand expression in multiple sclerosis

**DOI:** 10.1186/1742-2094-8-155

**Published:** 2011-11-08

**Authors:** Camille L Pittet, Jia Newcombe, Alexandre Prat, Nathalie Arbour

**Affiliations:** 1 Department of Medicine, Université de Montréal, CRCHUM, Pavilion J.A. de Sève, 1560 Sherbrooke E, Montreal, QC, H2L 4M1, Canada; 2 NeuroResource, UCL Institute of Neurology, 1 Wakefield Street, London WC1N 1PJ, UK; 3 Multiple Sclerosis Clinic, Centre Hospitalier Université de Montréal (CHUM)-Notre Dame Hospital, 1560 Sherbrooke E, Montreal, QC, H2L 4M1, Canada

**Keywords:** blood-brain barrier, CD8 T cells, endothelial cells, PD-L1, PD-L2, B7 molecules

## Abstract

**Background:**

Multiple sclerosis (MS), an inflammatory disease of the central nervous system (CNS), is characterized by blood-brain barrier (BBB) disruption and massive infiltration of activated immune cells. Engagement of programmed cell death-1 (PD-1) expressed on activated T cells with its ligands (PD-L1 and PD-L2) suppresses T cell responses. We recently demonstrated in MS lesions elevated PD-L1 expression by glial cells and absence of PD-1 on many infiltrating CD8 T cells. We have now investigated whether human brain endothelial cells (HBECs), which maintain the BBB, can express PD-L1 or PD-L2 and thereby modulate T cells.

**Methods:**

We used primary cultures of HBECs isolated from non-tumoral CNS tissue either under basal or inflamed conditions. We assessed the expression of PD-L1 and PD-L2 using qPCR and flow cytometry. Human CD8 T cells were isolated from peripheral blood of healthy donors and co-cultured with HBECs. Following co-culture with HBECs, proliferation and cytokine production by human CD8 T cells were measured by flow cytometry whereas transmigration was determined using a well established *in vitro *model of the BBB. The functional impact of PD-L1 and PD-L2 provided by HBECs was determined using blocking antibodies. We performed immunohistochemistry for the detection of PD-L1 or PD-L2 concurrently with caveolin-1 (a cell specific marker for endothelial cells) on post-mortem human brain tissues obtained from MS patients and normal controls.

**Results:**

Under basal culture conditions, PD-L2 is expressed on HBECs, whilst PD-L1 is not detected. Both ligands are up-regulated under inflammatory conditions. Blocking PD-L1 and PD-L2 leads to increased transmigration and enhanced responses by human CD8 T cells in co-culture assays. Similarly, PD-L1 and PD-L2 blockade significantly increases CD4 T cell transmigration. Brain endothelium in normal tissues and MS lesions does not express detectable PD-L1; in contrast, all blood vessels in normal brain tissues are PD-L2-positive, while only about 50% express PD-L2 in MS lesions.

**Conclusions:**

Our observations suggest that brain endothelial cells contribute to control T cell transmigration into the CNS and immune responses via PD-L2 expression. However, such impact is impaired in MS lesions due to downregulation of endothelium PD-L2 levels.

## Background

Multiple sclerosis (MS) is an inflammatory disorder of the central nervous system (CNS), pathologically characterized by focal demyelination, neuronal damage, glial cell activation and massive infiltration of immune cells [1]. Under physiological conditions, the blood-brain barrier (BBB) restricts and regulates the entrance of proteins, nutrients and cells from the periphery to the CNS [2,3]. However, during MS pathogenesis, the BBB impairment facilitates the infiltration of peripheral immune cells into the CNS [1]. Infiltrating cells detected within MS lesions include macrophages and T cells. Although CD4 T cells have been established as important players in MS pathogenesis, CD8 T cells are increasingly recognized as potential contributors to tissue damage [4,5]. CD8 T lymphocytes are detected in MS lesions, preferentially in the parenchyma and in greater numbers than their CD4 counterparts [6-11].

Programmed cell death-1 (PD-1), a member of the B7-CD28 family, is a co-inhibitory receptor expressed by a variety of activated immune cells, including T cells [12]. The interaction between PD-1 and its ligands (PD-L1 or PD-L2) suppresses T cell responses including proliferation, cytokine production, and cytotoxicity [12-15]. PD-L1 is expressed by activated immune cells [16] such as T cells, B cells, macrophages, dendritic cells and microglia [17], as well as by non-immune cells such as endothelial and epithelial cells [18,19], and astrocytes [17]. PD-L2 expression is more restricted and has been observed on macrophages, dendritic cells, mast cells [16], and endothelial cells from various organs [15,20-23]. Several groups have established that PD-L1 and PD-L2 expression varies between different endothelial sources and species (mouse *vs*. human) and that such expression displays immuno-regulatory functions [15,20,21,23]. However, whether human brain endothelial cells (HBECs) via the expression of PD-L1 and/or PD-L2 impact on immune responses has not been investigated.

Studies performed in the experimental autoimmune encephalomyelitis (EAE) mouse model of MS have underlined the contribution of PD-1 and its ligands to dampening disease susceptibility or severity [24-26]. Moreover, blocking PD-1 using antibodies or knock-out mice led to an elevated number of CNS infiltrating immune cells, especially CD8 T cells [25-27]. We have previously shown [17] that although PD-L1 is barely detectable in the brain of normal controls, its expression is significantly increased in MS lesions, especially on astrocytes and microglia/macrophages [17]. We observed that although the very few CD8 T cells found in control brain are all PD-1 positive, the majority of infiltrating CD8 T cells in MS lesions do not express PD-1. Whether T cell infiltration into the inflamed CNS of MS patients is modulated by the BBB via the expression of PD-L1 and/or PD-L2 is still unresolved.

In this study, we investigated PD-L1 and PD-L2 expression by primary cultures of HBECs and the impact of such expression on CD8 T cell functions. We demonstrate that HBECs express low/undetectable levels of PD-L1 at basal level *in vitro*, but most of them express PD-L2 and both ligands are up-regulated in response to pro-inflammatory stimuli. Moreover, we establish that via the expression of PD-L1 and PD-L2, HBECs can locally modulate human T cell responses, leading to decreased migration of CD8 and CD4 T cells through an *in vitro *BBB model. Finally, we assessed the expression of PD-L1 and PD-L2 in post-mortem human brain tissues. Whereas PD-L1 is not detectable on endothelial cells in control or MS tissue sections, PD-L2 is robustly detected in control brain tissues but its expression is partially lost in MS lesions.

## Methods

### Isolation and culture of human brain endothelial cells

CNS tissue was obtained from surgical resections performed for the treatment of non-tumor related intractable epilepsy as previously described [28]. Consent and ethical approval were given prior to surgery (BH 07.001). Human brain endothelial cells (HBECs) were grown in M199 medium (Invitrogen, Burlington, ON, Canada) supplemented with 10% fetal bovine serum, 20% normal human serum, endothelial cell growth supplement (5 μg/ml) and insulin-selenium-transferin premix on 0.5% gelatin-coated tissue culture plates (all reagents from Sigma, Oakville, ON, Canada).

### Isolation of human T cells

A written informed consent was obtained from healthy donors in accordance with the local ethical committee (HD 07.002 and BH 07.001). Peripheral blood mononuclear cells (PBMCs) were obtained by Ficoll density gradient as previously described [29]. CD8 or CD4 T cells were positively isolated from PBMCs using either CD8 or CD4 beads respectively (MACS, Miltenyi Biotec, Auburn, CA, USA) according to the manufacturer's instructions; purity assessed by flow cytometry was typically > 95%.

### RNA isolation, reverse transcription, and qPCR

Total RNA was extracted and transcribed into cDNA as previously described [17,30]. Relative mRNA expression was determined by quantitative real-time PCR (qPCR) using primers and TaqMan FAM-labeled MGB probes for PD-L1 and PD-L2 and ribosomal 18S (VIC-labeled probe, used as an endogenous control) obtained from Applied Biosystems (Foster City, CA, USA) according to manufacturer's instructions and as previously described [17,30].

### Flow cytometry

Cells were stained for surface and/or intracellular molecules as previously described [17,31], acquired on a LSRII (BD Biosciences, Mississauga, ON, Canada) and analyzed with FlowJo software (Treestar, Ashland, OR, USA). Mouse monoclonal antibodies directed at human protein and conjugated to biotin, fluoroscein isothiocyanate (FITC), Alexa Fluor^® ^700, phycoerythrin, Pacific Blue, or allophycocyanin were used. Surface stainings targeted: PD-L1 (eBioscience, San Diego, CA, USA), PD-L2 (eBioscience), HLA-ABC (Biolegend, San Diego, CA, USA), CD4 and CD8 (BD Biosciences). Intracellular stainings targeted: granzyme B (Caltag, Buckingham, UK) and IFN-γ (BD Biosciences). Appropriate isotype controls were used for all stainings. Δmedian fluorescence intensity (ΔMFI) was calculated by subtracting the fluorescence of the isotype from that of the stain.

### Migration assay

Migration assays were performed in a modified Boyden chamber as previously described [28]. HBECs plated on Boyden chambers (Collaborative Biomedical Products, Bedford, MA) were stimulated with IFN-γ (200 U/ml) and TNF (200 U/ml) for 24 hours and then treated either with isotype control antibodies or blocking antibodies specific for PD-L1 (10 μg/ml, eBioscience) and/or PD-L2 (10 μg/ml, eBioscience) for one hour at 37°C. CD8 or CD4 T cells that had been exposed to plate-bound anti-CD3 (0.9 μg/ml, clone OKT3, purified in house) and anti-CD28 antibodies (1 μg/ml, BD Biosciences) for 72 hours were then added to the upper chamber (1 × 10^6 ^cells per Boyden chamber) and allowed to migrate for 24 hours across HBECs. FITC-labeled BSA (50 μg/ml; Invitrogen) was concurrently added to the upper chamber and 50 μl samples were harvested from the upper and lower chambers at different time points and the fluorescence intensity in these samples was measured using a Synergy4 Biotek microplate reader. The diffusion rate of the FITC-BSA, a measure of the permeability, was expressed as a percentage and calculated as followed: [(BSA lower chamber)/(BSA upper chamber)] × 100. After migration, cells from the lower and upper chambers were collected, counted, and stained for different markers.

### Co-culture assay

HBECs were plated (5 × 10^5 ^cells per well in a 24-well plate), and after 3 days when reaching confluence, stimulated with IFN-γ (200 U/ml) and TNF (200 U/ml) for 24 hours. HBECs were washed three times to remove these inflammatory cytokines. An isotype control antibody or blocking antibodies specific for PD-L1 (10 μg/ml) and/or PD-L2 (10 μg/ml) were added one hour at 37°C to allow them to bind to their cognate ligands. Isolated alloreactive human CD8 T cells were labeled with CFSE as previously described [29] and then subsequently added (2 × 10^5 ^cells per well) to HBECs without removing the blocking antibodies and in the presence of anti-CD3 (0.18 μg/ml) and anti-CD28 (1 μg/ml) antibodies. Blocking antibodies and anti-CD3 and anti-CD28 were left in the wells for the entire co-culture. After a 6 day co-culture, CD8 T cells were collected and stained for Live/dead fixable Aqua dead cell stain kit (Invitrogen) to exclude dead cells and stained for CD8, granzyme B, and IFN-γ for flow cytometry assessment.

### Immunohistochemistry

Post-mortem brain sections from tissue donors without CNS disease and patients diagnosed clinically and confirmed by neuropathological examination as having MS were obtained from the NeuroResource tissue bank, UCL Institute of Neurology, London, U.K. Tissues were donated to the tissue bank with informed consent following ethical review by the London Research Ethics Committee, UK. This study was approved by the CHUM Ethical Committee (HD 07.002). Snap-frozen coded sections (~1 cm^2 ^and 10 μm thick) were cut from blocks of normal control and MS brain tissues. Sections cut before and immediately after the ones used for the immunofluorescence studies were stained with oil red O and hematoxylin, and scored as previously described [32] (Table 1). Sections were air-dried, fixed in cold acetone for 10 min, and blocked for non-specific binding for 1 hour with 10% donkey (for PD-L2 detection) or goat serum (for PD-L1 detection). Primary antibodies targeting PD-L1 (25 μg/ml, Biolegend) or PD-L2 (2 μg/ml, RD Systems, Burlington, ON, Canada) was incubated 1 hour at room temperature and then overnight at 4°C. Sections were then washed with PBS and incubated for 40 minutes with appropriate secondary antibodies: Alexa Fluor^® ^488-conjugated goat-anti-mouse for PD-L1 and Alexa Fluor^® ^488-conjugated donkey-anti-goat for PD-L2. Sections were then incubated at room temperature for 1 hour with antibodies targeting cell specific markers for endothelial cells (rabbit-anti-human-caveolin-1, Santa Cruz Biotechnology, Santa Cruz, CA, USA) followed by 40 minutes with secondary antibody (Rhodamine-conjugated goat-anti-rabbit, Jackson Immunoresearch, West Grove, PA, USA). Finally, sections were incubated with a nuclear stain TO-PRO^®^-3 iodide (Invitrogen), treated with Sudan Black and mounted as previously described [17]. Controls were concurrently carried out on adjacent sections using appropriate primary isotype controls at the same concentrations. Slides were observed using a SP5 Leica confocal microscope. Confocal images were acquired simultaneously in different channels throughout 4-8 μm z-stack every 0.2-0.5 μm. We validated staining specificity by lack of signal only when the corresponding laser was turned off but not when others were still on. Several fields (> 5) containing blood vessels were taken randomly on each section and used for quantification of positive cells. Moreover, we confirmed the absence of bleed-through by re-examining selected sections using sequential scanning.

**Table 1:** Description of post-mortem brain sections

Block	M/F	Age (Y)	DD (Y)	Cause of death	DFT (h)	Sample Type	ORO, hematoxylin Score	Summary observations on ORO-stained sections
1	F	68	-	Colo-rectal metastatic tumour	23	NC W, OV, R	0, 0	Normal white matter and cortical grey matter

2	M	49	-	Myocardial infarction and coronary artery thrombosis	11	NC W, PV, R	0, 0	Normal white matter and grey matter.

3	M	53	-	Cardiac arrest	19	NC W, OSv, R	0, 0	Normal white matter.

4	F	47	20	Bronchopneumonia	9	MS AQ, FSv, L	4, 3	White matter and grey matter surrounding active plaque. ORO+ cells in blood vessel walls and parenchyma.

5	F	47	20	Bronchopneumonia	9	MS AQ, PSv, L	5, 4	Large plaque with active and some subacute and chronic areas. Large perivascular cuffs. White and grey matter.

6	F	37	10	Bronchopneumonia	24	MS AQ, basal ganglia, L	3, 3	Large subacute plaque with perivascular cuffing; areas of grey matter.

7	F	71	32	Bronchopneumonia	19	MS SAQ, O pole Sv, L	2, 0	Hypocellular plaque surrounded by patchy abnormal-appearing white matter.

8	F	29	8	Bronchopneumonia	11	MS SAQ, cerebellum, R	1, 1	Large subacute plaque with hypercellular areas.

9	F	60	34	Renal failure	24	MS SAQ, TV, L	2, 4	Large subacute plaque with many large and small perivascular cuffs.

10	F	49	11	Bronchopneumonia	16	MS CQ, F pole V, R	0, 2	Large chronic plaque surrounded by pale abnormal white matter.

Abbreviations:

DD: disease duration; DFT: time between death and sample freezing; NC: normal control; W: white matter; MS: multiple sclerosis; AQ: active plaque; SAQ: subacute plaque; V: ventricular; Sv: subventricular; F: frontal; P: parietal; T: temporal; O: occipital; R: right; L: left.

ORO and cuffing: Scored on a scale of 0 to 5 for ORO and haematoxylin staining; 0 is what would be expected in normal control white matter. Data is averaged from duplicate sections cut immediately before and after the serial sections cut.

### Statistical analyses

Statistical analyses were performed using PRISM Graphpad™ software (La Jolla, CA, USA) and included Student's *t*-test; *P*-values < 0.05 were considered significant.

## Results

### Pro-inflammatory cytokines increase PD-L1 and PD-L2 expression by human brain endothelial cells

**Figure 1:**
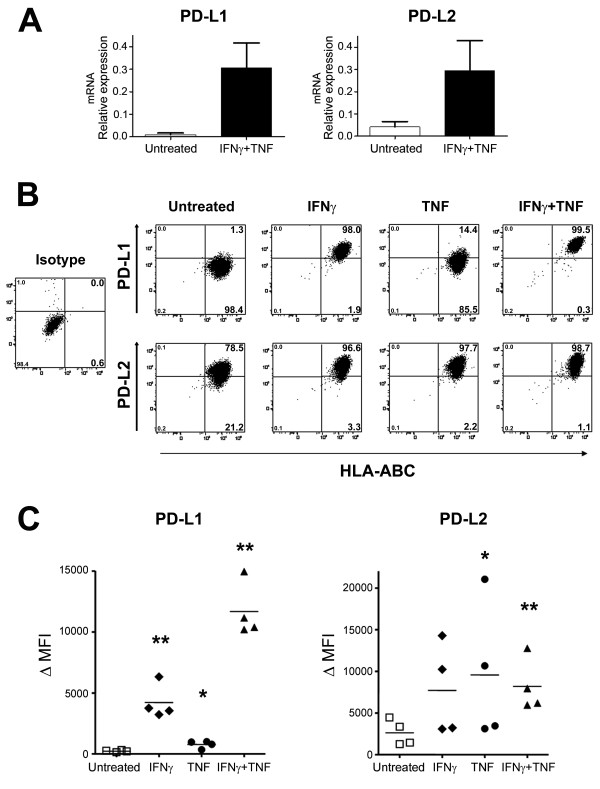
**Pro-inflammatory stimulation increases PD-L1 and PD-L2 expression on human brain endothelial cells**. Human brain endothelial cells were either left untreated or stimulated with inflammatory cytokines as indicated and then PD-L1 and PD-L2 expression was determined by qPCR and flow cytometry. A. Pooled data (n = 4) of PD-L1 and PD-L2 mRNA relative levels in response to IFN-γ+TNF. B. Representative flow cytometry dot plots illustrating PD-L1 and PD-L2 protein expression versus HLA-ABC expression in response to different cytokines. C. PD-L1 (left panel) and PD-L2 (right panel) protein expression (ΔMFI) observed for 4 distinct donors. Student's t-test: * *P *< 0.05, ** *P *< 0.01.

We evaluated whether HBECs express detectable levels of PD-L1 and/or PD-L2. Primary cultures of HBECs were either left untreated or activated with pro-inflammatory cytokines IFN-γ, TNF, or IFN-γ+TNF to mimic the pro-inflammatory environment typically observed in the CNS of MS patients. HBECs expressed very low PD-L1 but detectable PD-L2 mRNA levels under basal conditions as assessed by qPCR (Figure 1A). IFN-γ+TNF treatment robustly increased those levels (Figure 1A), almost reaching statistical significance for PD-L1 expression (n = 4 donors, untreated vs. IFN-γ+TNF p = 0.075). Detection of PD-L1 and PD-L2 proteins by flow cytometry allowed quantification of both percentages of HBECs expressing these molecules and intensity of such expression (ΔMFI); typical flow cytometry detection is shown (Figure 1B). HBECs under basal conditions expressed very low/undetectable levels of PD-L1 protein (Figure 1B: 1.3%), while PD-L2 protein was already expressed by the majority of cells reaching 78.5% (Figure 1B). In response to different cytokine treatments tested, the proportion of HBECs expressing PD-L1 significantly increased reaching over 96%, especially in response to IFN-γ and IFN-γ+TNF (Figure 1B) (mean n = 4, PD-L1+ cells: IFN-γ: 98.5 ± 1.2% and IFN-γ+TNF 99.4 ± 0.3%; ** p < 0.003 compared to untreated), while TNF (Figure 1B) had a more modest impact (mean n = 4, PD-L1+ cells: 54.1 ± 15.6%, p = 0.065 compared to untreated). All cytokine treatments tested also boosted the proportion of HBECs expressing PD-L2 reaching over 96% (mean n = 4, PD-L2+ cells: IFN-γ: 97.2 ± 1.4%; IFN-γ+TNF: 98.9 ± 0.6%; TNF: 97.5 ± 2.4%). Moreover, cytokine treatments led to not only increased proportions of HBECs expressing PD-L1 or PD-L2 but also elevated intensity as shown by ΔMFI; IFN-γ+TNF having the more potent impact for PD-L1 levels (Figure 1C). We also observed an upregulation of MHC-class I molecules (HLA-ABC, Figure 1B) on HBECs upon cytokine treatment. In agreement with our flow cytometry results, PD-L1 was undetectable by immunocytochemistry on untreated HBECs but reached detectable levels after IFN-γ+TNF treatment, while PD-L2 was detectable both under basal conditions and following pro-inflammatory treatments (data not shown).

### Human brain endothelial cells partially block T cell migration through an *in vitro *model of the BBB via PD-L1 and PD-L2

**Figure 2:**
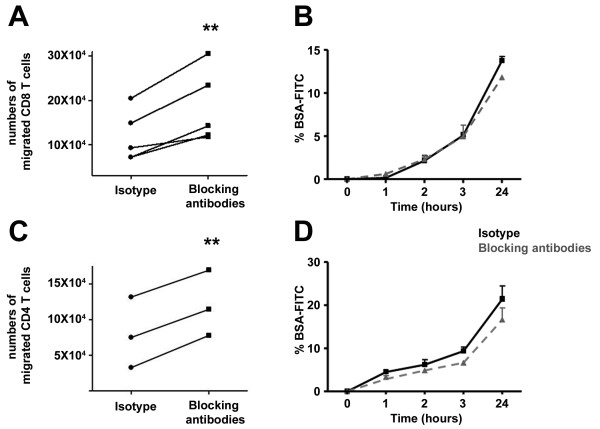
**Blocking PD-L1/2 enhances migration of CD8 and CD4 T cells through an *in vitro *BBB model**. HBECs were plated to the upper chamber of a Boyden chamber and then inflamed. Activated CD8 (A, B) and CD4 (C, D) T cells were added to the upper chamber and allowed to migrate for 24 hours in the presence of an isotype control antibody or blocking antibodies specific for PD-L1 and PD-L2. A-C. Graphs representing the number of CD8 (A) or CD4 (C) T cells migrating through the *in vitro *BBB for 3-5 distinct T cell donors on 2-3 HBECs preparations. Student's t-test: ** *P *< 0.01. B-D. Graphs representing the percentage of BSA-FITC diffusion as a measure of permeability of the *in vitro *BBB, showing one representative experiment out of three.

We elected to address whether the expression of PD-L1 and PD-L2 by HBECs influences their capacity to regulate the migration of CD8 and CD4 T cells into the CNS. We used a well-established *in vitro *model of the BBB in which HBECs are seeded in the upper compartment of a Boyden chamber [33], inflamed, and then incubated with either anti-PD-L1 and anti-PD-L2 blocking antibodies or isotype control antibodies. CD8 or CD4 T cells that have been stimulated for 3 days with anti-CD3+anti-CD28 to maximally increase the expression of PD-1 were added to the Boyden chamber. We have previously shown that although on average only 14% of *ex vivo *CD8 T cells expressed PD-1, this proportion reached 64% after such a stimulation [17]. We observed significantly greater numbers of CD8 T cells migrating through the *in vitro *BBB when blocking antibodies targeting PD-L1+PD-L2 were added compared to the isotype control (Figure 2A). Blocking only one ligand PD-L1 or PD-L2 had a more modest impact on the number of migrated CD8 T cells (data not shown). In parallel, we performed a permeability assay using BSA-FITC as a permeability tracer and observed an identical diffusion of BSA-FITC for the isotype control and the blocking antibodies conditions (Figure 2B). These results demonstrate that the elevated CD8 T cells transmigrating through the *in vitro *BBB in the presence of anti-PD-L1+anti-PD-L2 blocking antibodies were not due to a general disruption of the brain endothelial cell monolayer. Similarly, blocking these ligands led to an increased number of CD4 T cells migrating through our *in vitro *BBB (Figure 2C-D). Therefore, PD-L1 and PD-L2 expressed by HBECs contribute to dampening T cell migration through the barrier created by these specialized cells.

### Human brain endothelial cells modulate T cell responses via PD-L1 and PD-L2

**Figure 3:**
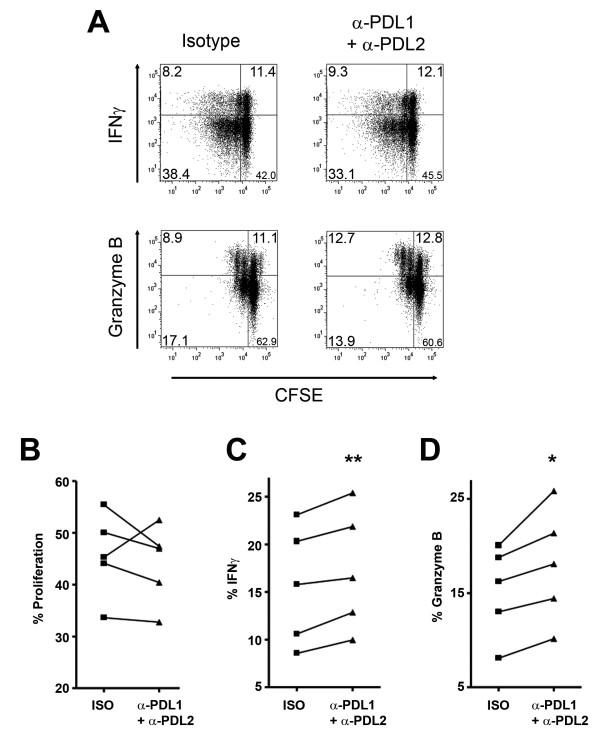
**Expression of PD-L1 and PD-L2 on HBECs regulates CD8 T cell responses**. HBECs were first stimulated with IFN-γ and TNF for 24 hours. After three washes, HBECs were incubated either with an isotype control antibody or blocking antibodies specific for PD-L1 and PD-L2, prior to the addition of *ex vivo *human CD8 T cells labeled with CFSE in the presence of anti-CD3 and anti-CD28. These reagents were left for the entire co-culture period. After 6 days of co-culture, proliferation, IFN-γ and granzyme B were analyzed by flow cytometry. A. Representative dot plots of CD8 T cell responses for 2 different donors: proliferation assessed by CFSE dilution (X axis) vs. IFN-γ (top panel) or granzyme B (bottom panel) production (Y axis). Flow cytometry plots are gated on living CD8 T cells. B-D. Data obtained from 5 CD8 T cell donors on 3 HBECs preparations for proliferation (B), IFN-γ (C) and granzyme B production (D). Student's t-test: * *P *< 0.05, ** *P *< 0.01.

Previous studies have shown the capacity of PD-L1 and PD-L2 expressing endothelial cells, especially human umbilical vein endothelial cells (HUVECs) and mouse cardiac endothelial cells, to inhibit T cell responses [15,20-23]. However, whether human brain endothelial cells could modulate CD8 T cells responses has not been previously determined. Alloreactive human CD8 T cells were labeled with CFSE and then added to inflamed HBECs cultures that have been pre-incubated with either isotype control antibodies or anti-PD-L1 and anti-PD-L2 blocking antibodies. Proliferation (CFSE low cells) and production of IFN-γ and granzyme B were analyzed by flow cytometry. As shown for 2 different donors in Figure 3, blocking PD-L1 and PD-L2 had no consistent effect on CD8 T cell proliferation (Figure 3A, B). On the other hand, a modest but significant increase in the percentage of IFN-γ producing CD8 T cells (Figure 3A, C) was observed for all donors when PD-L1 and PD-L2 were blocked compared to the isotype control. The percentage of granzyme B producing CD8 T cells (Figure 3A, D) was significantly elevated when both ligands were blocked compared to the isotype control. Blocking only PD-L1 or only PD-L2 led to a partial increase of IFN-γ and granzyme B production in comparison to the blockage of both proteins (data not shown). Our results demonstrate that PD-L1 and PD-L2 expressed by HBECs were sufficient to significantly diminish the effector functions (cytokines and lytic enzyme) of human CD8 T cells.

### Human brain endothelial cells in MS lesions do not express PD-L1, while PD-L2 is down-regulated

**Figure 4:**
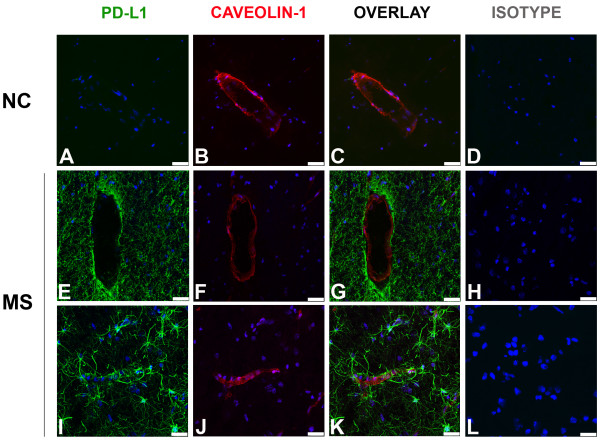
**PD-L1 is not detectable on brain endothelium of normal controls and MS patients**. Micrographs showing brain sections stained for PD-L1 (green), caveolin-1 (red) and nucleus (blue) of one representative normal control (A-C) and two representative MS donors (E-G, I-K). In control subjects, PD-L1 immunoreactivity is not detectable (A). In contrast, PD-L1 is robustly expressed in MS lesions but it does not co-localize with the endothelial cell marker (E-G, I-K). Corresponding isotypes are shown in D, H and L. Scale bar: 25 μm.

**Figure 5:**
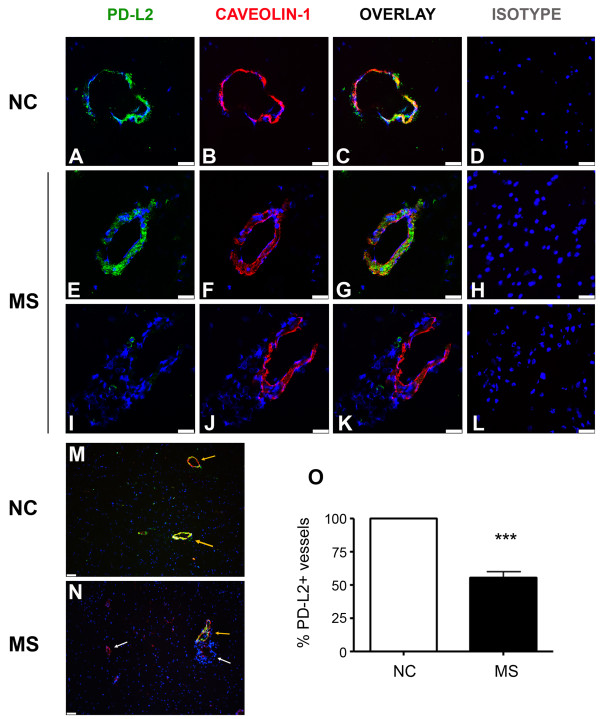
**PD-L2 is expressed on brain endothelial cells of normal controls, but down-regulated in MS lesions**. Micrographs showing brain sections stained for PD-L2 (green), caveolin-1 (red) and nucleus (blue) of one representative normal control (A-C, M) and two representative MS donors (E-G, I-K, N). PD-L2 immunoreactivity is detected on all blood vessels visualized by caveolin-1 positive labeling in control subjects (C, M). However, in MS lesions, while PD-L2 reactivity is detected on blood vessels (E-G, N), a subset of blood vessels visualized by caveolin-1 labeling does not express detectable PD-L2 (I-K, N). Corresponding isotypes are shown in D, H and L. Scale bar: 25 μm (A-L), 400 μm (M, N). White arrows indicate examples of caveolin-1+ PD-L2- blood vessels, whereas orange arrows indicate examples of caveolin-1+ PD-L2+. O. Quantification of the percentage of blood vessels expressing PD-L2 in sections from normal controls (n = 3) and MS patients (n = 7). At least 6 blood vessels were counted for each section. Student's t-test: *** *P *< 0.001.

To assess whether endothelial cells in the CNS of MS patients express PD-L1 and/or PD-L2, we performed immunohistochemistry on post-mortem brain tissues obtained from normal controls and MS patients (see description in Table 1). MS lesions were characterized using oil red 0 (ORO) and hematoxylin scoring as being acute, containing numerous phagocytic macrophages that had recently engulfed lipid-containing debris, or subacute, containing demyelinated areas but demonstrating less recent myelin destruction. Brain sections were stained for PD-L1 or PD-L2 and caveolin-1, a specific marker for endothelial cells, or appropriate isotype controls. Six to ten fields (at 630×, each field covering 0.0625 mm^2^) per section containing caveolin-1+ blood vessels were selected randomly (3 sections from controls and 7 sections MS lesions) and thoroughly analyzed to determine the percentage of blood vessels positive for PD-L1 or PD-L2, and representative fields are illustrated (Figures 4, 5). As shown in our earlier study [17], no or very low expression of PD-L1 was observed in the CNS of normal controls (Figure 4A-C). However, as we have previously reported, an elevated expression of PD-L1 was observed on astrocytes and microglia/macrophages in MS lesions, but no co-localization was found between PD-L1+ cells and caveolin-1+ cells (Figure 4E-G and 4I-K).

In contrast to PD-L1, PD-L2 was easily detected in normal control brain sections, and was co-localized with caveolin-1+ cells (Figure 5A-C, M). Whereas all caveolin-1+ cells were positive for PD-L2 in normal control sections, PD-L2 was only expressed by a subset of endothelial cells in MS sections (Figure 5E-G, I-K, N). Quantification of blood vessels identified by caveolin-1 labeling demonstrated that all blood vessels in normal tissues expressed PD-L2 but only 50% expressed PD-L2 in MS lesions (Figure 5O). The reduced PD-L2 expression was observed on vessels of all diameters and regardless whether these lesions were acute, sub-acute, or chronic. However, diminished PD-L2 expression was localized within areas of demyelination and not outside MS lesions as assessed by Sudan black staining [17]. PD-L2 labeling was either easily detectable or absent (Figure 5). PD-L2+ but caveolin-negative cells with a morphology suggestive of infiltrating leukocytes were observed around some blood vessels in MS lesions, whereas outside lesions and in normal control sections these cells were not seen.

## Discussion

In this study, we demonstrate that primary cultures of HBECs express robust basal levels of PD-L2 and increased levels of PD-L1 and PD-L2 in response to pro-inflammatory cytokines. Such PD-1 ligand expression contributes to the capacity of HBECs to reduce the migration and activation of human T cells. Our analysis of post-mortem human brain tissues underlines that PD-L2 is expressed by all brain endothelial cells under normal physiological conditions but that a significant proportion of these cells do not express PD-L2 in MS brain lesions. Finally, PD-L1 although easily observed on other CNS cell types in MS brain lesions is not detected on brain endothelial cells.

PD-L1 and PD-L2 expression by endothelial cells from various origins, but not CNS, has been previously shown. Using primary cultures of HBECs, we observed that under physiological conditions PD-L1 was not detected as assessed by flow cytometry and qPCR. On the other hand, PD-L2 was already highly expressed at basal level (Figure 1). Upon inflammation, both ligands were up-regulated, reaching around 100% of cells positive for these ligands (Figure 1). Previous studies have shown similar observations using human umbilical vein endothelial cells (HUVECs) by qPCR and flow cytometry: HUVECs did not express PD-L1 [15,22,23], but did bear considerable PD-L2 levels under basal conditions [15]. Moreover, IFN-γ stimulation increased both PD-L1 and PD-L2 expression while the combination IFN-γ+TNF was even more potent [15]. Also using flow cytometry, human cornea endothelial cells increased PD-L1 and PD-L2 levels following IFN-γ stimulation [20]. In contrast, mouse heart endothelial cells upregulated PD-L1 levels in inflammatory conditions but did not express detectable levels of PD-L2 under basal or activated conditions as assessed by flow cytometry and microscopy [15,21], suggesting distinct regulation of PD-L1 and PD-L2 by endothelial cells in different species and in different organs.

Massive infiltration of immune cells into the CNS is one of the first steps leading to the formation of new MS lesions and mechanisms controlling such infiltration have not been completely elucidated. Blocking PD-L1 and PD-L2 in EAE, the mouse model of MS, leads to earlier onset and increased severity of the disease, mainly due to elevated number of infiltrating immune cells, especially CD8 T cells [25,26]. In our study, we demonstrated that blocking PD-L1 and PD-L2 on HBECs leads to elevated number of CD8 and CD4 T cells migrating through an *in vitro *BBB model (Figure 2), supporting a contributing role for these ligands expressed by the local endothelium in regulating immune cell infiltration into the CNS. In contrast, our group has recently shown that MHC class I blockade does not modify the migration of human CD8 T cells across BBB-endothelial cells [34]. These observations also demonstrate that although CD8 T cells and HBECs were obtained from different human donors, the allo-reactivity did not play a role in CD8 T cell migration in our *in vitro *BBB model. Furthermore, it has been previously demonstrated that the ligation of PD-1 blocks the β1 and β2 integrin-mediated adhesion by human T cells induced with anti-CD3 [35]. Therefore, based on these published data and our own novel data, we suggest that the binding of PD-1 on T cells by PD-L1/2 on HBECs prevents these T cells from crossing the endothelium potentially via a mechanism implicating integrins. CD8 T cells were shown to be particularly affected by a general PD-L1 and PD-L2 blockade in the EAE model [26,27]. We can speculate that PD-1 ligand expression by the CNS-endothelium may play a role in regulating the migration of other activated immune cells expressing the cognate receptor, as PD-1 is expressed not only on activated T cells but also on B cells and monocytes [36].

Endothelial cells from different organs have been shown to display the capacity to modulate T cell responses via the expression of PD-L1 and/or PD-L2 [15,21-23]. In our studies, blocking PD-L1 and PD-L2 on inflamed HBECs did not affect the proliferation of CD8 T cells. However, it had an impact on the production of IFN-γ and granzyme B (Figure 3). Rodig and colleagues have similarly demonstrated that blocking PD-L1 and/or PD-L2 on HUVECs increased the production of IFN-γ, but did not influence proliferation and IL-2 production by CD8 T cells [15]. This group also reported that blocking PD-L1 on mouse heart endothelial cells increase the killing capacity of CD8 T cells. We believe that the effects seen in our *in vitro *assays were mainly due to the blocking of PD-L1 and PD-L2 on inflamed HBECs but we cannot rule out that the anti-PD-L1 antibody could bind to PD-L1-expressing activated CD8 T cells. However, as we could not detect PD-L1 on *ex vivo *CD8 T cells and only low levels of PD-L1 on a small fraction of CD8 T cells (8-20%) after anti-CD3+anti-CD28 activation (data not shown), this would be a less important contribution.

Distinct PD-L1 and PD-L2 expression has been reported in different human organs. Several groups demonstrated that PD-L1 and/or PD-L2 are detected in immuno-privileged organs under physiological conditions. PD-L1 is elevated in human placenta, while PD-L2 is highly expressed on the endothelium of placenta blood vessels [37]. Although PD-L1 is constitutively expressed in testis, another immuno-privileged organ, no PD-L2 is observed [38]. PD-L1 is also constitutively expressed at high levels by corneal epithelial cells. However, these cells bear significantly reduced PD-L1 levels during dry eye disease, a T-cell mediated inflammation [39], paralleling our observations for PD-L2 on human CNS endothelium in controls vs. MS. Using an endothelial cell specific marker (caveolin-1), we easily detected PD-L2 expression by all blood vessels (caveolin-1+) in post-mortem CNS tissues obtained from normal controls, but only on about 50% of blood vessels in MS lesions (Figure 5). We observed non-endothelial cells around blood vessels expressing PD-L2 in MS lesions. According to the shape and the localization of these cells, we hypothesize that these are infiltrating immune cells. Experiments performed in EAE documented PD-L1 and PD-L2 detection on a fraction of infiltrating immune cells such as macrophages, dendritic cells and microglia [26,40]. We could not detect PD-L1 on CNS brain endothelium although this ligand was easily observed on other CNS cells in MS lesions (Figure 4) and has been observed on malignant gliomas [41]. We have previously shown that PD-L1 is significantly elevated in MS brain lesions especially on astrocytes and microglia/macrophages [17], while this ligand is barely detectable in normal controls. These observations correlate with our previous *in vitro *data obtained with primary cultures of glial cells; we detected low levels of PD-L1 on microglia and astrocytes under basal conditions but a significant increase of PD-L1 levels on these cells upon pro-inflammatory stimulation [17]. In contrast to our observations in human CNS, PD-L2 was not detected on CNS cells of control and EAE animals, although PD-L1 was observed on resident brain cells, including the endothelium, in EAE mice [26,42]. These results support the notion that PD-L1 and PD-L2 expression is differently regulated in human and murine CNS [42].

Recent work suggests that the expression of PD-1 ligands is regulated by different promoters in distinct cell types [43]. Indeed, whereas murine PD-L2 expression has been shown to be controlled by NF-κB and STAT6, PD-L1 expression is not [42]. Moreover, platinum-based chemotherapeutics have been shown to downregulate PD-L2 expression in human dendritic and tumor cells [44] via a STAT6-mediated mechanism. Therefore, a more detailed dissection of the mechanisms regulating PD-L1 and PD-L2 expression under physiological and disease conditions is warranted and could result in new therapeutic tools.

Our *in vitro *data showed that HBECs express low or no PD-L1 but high PD-L2 levels under basal conditions; similarly in normal control brain tissues we did not detect PD-L1 but observed robust PD-L2 expression by the brain endothelium. Although inflammatory cytokines increased PD-L1 and PD-L2 levels *in vitro*, these ligands were not upregulated in MS lesions compared to controls. In contrast to glial cells, endothelial cells are sitting at the boundary between the periphery and the CNS. We can hypothesize that factors, others than pro-inflammatory cytokines, present in the periphery on the lumen side, or other CNS cells closely interacting with the endothelium, may impact on the *in vivo *PD-L1 and PD-L2 expression by the CNS endothelium. Finally, we can speculate that under physiological conditions, the elevated PD-L2 basal levels contribute to inhibit the activation and migration of T cells across the BBB, but given the reduced levels of PD-L2 on MS brain endothelium, this function is impaired. CD8 T cells have been reported to be localized more frequently in the parenchyma of MS brain [8,9,45]. Furthermore, we observed an important PD-L1 upregulation [17] in MS lesions in perivascular and parenchymal areas, correlating with the absence of PD-1 on infiltrating CD8 T cells. Therefore, we speculate that the BBB capacity to control cell entry into the CNS is impaired in MS patients, leading to the entry of T cells regardless whether they express PD-1 or not, but that PD-1-negative CD8 T cells will be favored for progressing into the inflamed parenchyma which abundantly expresses PD-L1.

## List of abbreviations

BBB: blood brain barrier; CNS: central nervous system; EAE: experimental autoimmune encephalomyelitis; HBECs: human brain endothelial cells; HUVECs: human umbilical vein endothelial cells; ΔMFI: delta median fluorescence intensity; MS: multiple sclerosis; PBMCs: peripheral blood mononuclear cells; PD-1: programmed cell death-1; PD-L1: programmed cell death-ligand 1; PD-L2: programmed cell death-ligand 2.

## Competing interests

The authors declare that they have no competing interests.

## Authors' contributions

CLP conducted most of the experiments; JN provided human brain sections, performed pathological scoring and provided input on the manuscript; AP provided the primary cultures of HBECs and relevant expertise on the transmigration assays; CLP and NA designed the study, analyzed the data and wrote the manuscript; NA secured the funding. All authors read and approved the final manuscript.
